# Mutations in Coagulation *Factor VIII* Are Associated with More Favorable Outcome in Patients with Cutaneous Melanoma

**DOI:** 10.1055/s-0037-1607337

**Published:** 2017-10-20

**Authors:** Zheng Ping, Abha Soni, Lance A. Williams, Huy P. Pham, Malay K. Basu, X. Long Zheng

**Affiliations:** 1 Division of Laboratory Medicine, The University of Alabama at Birmingham, Alabama, United States; 2 Division of Informatics, Department of Pathology, The University of Alabama at Birmingham, Alabama, United States

**Keywords:** coagulation factors, tumor metastasis, factor VIII, von Willebrand factor, ADAMS/ADAMTS13

## Abstract

Coagulation factor VIII (FVIII), von Willebrand factor (VWF), and ADAMTS13 (a disintegrin and metalloprotease with thrombospondin type 1 repeats 13) play an important role in the regulation of normal hemostasis. However, little is known about their roles in patients with malignancy, particularly with cutaneous melanoma. Whole genome sequencing data are available for 25,719 cases in 126 cancer genomic studies for analysis. All sequencing data and corresponding pathology findings were obtained from The Cancer Genome Atlas. The cBioPortal bioinformatics tools were used for the data analysis. Our results demonstrated that mutations in genes encoding *FVIII* , *VWF* , and *ADAMTS13* were reported in 92 of 126 cancer genomic studies, and high mutation rates in these three genes were observed in patients with cutaneous melanoma from three independent studies. Moreover, high mutation rates in *FVIII* , *VWF* , and *ADAMTS13* were also found in patients with diffuse large B cell lymphoma (22.9%), lung small cell carcinoma (20.7%), and colon adenocarcinoma (19.4%). Among 366 melanoma cases from TCGA provisional, the somatic mutation rates of *FVIII* , *VWF* , and *ADAMTS13* in tumor cells were 15, 14, and 5%, respectively. There was a strong tendency for coexisting mutations of *FVIII* , *VWF* , and *ADAMTS13* . Kaplan–Meier survival analysis demonstrated that melanoma patients with *FVIII* mutations had a more favorable overall survival rate than those without *FVIII* mutations ( *p* = 0.02). These findings suggest, for the first time, that the *FVIII* mutation burden may have a prognostic value for patients with cutaneous melanoma. Further studies are warranted to delineate the molecular mechanisms underlying the favorable prognosis associated with *FVIII* mutations.

## Introduction

The link between coagulation and malignancy has been recognized for over a century. 1 2 Thrombosis is a common cause of death in cancer patients. Pulmonary embolism (PE) was more commonly detected in cancer patients at autopsy than in those without malignancy. 3 PE may also be the presenting signs of an underlying occult malignancy. 4 In addition, it is accepted that coagulation activation and platelet activation in the tumor microenvironment may have a biological significance affecting tumor growth and dissemination (or metastasis).

Cutaneous melanoma is a common skin cancer with an incidence rate of 14.1 per 100,000 inhabitants per year, 5 arising from the pigment-containing cells known as melanocytes. Ultraviolet radiation from sunlight or tanning devices can induce malignant transformation of these cells by inducing DNA damage, especially in fair-skinned individuals. In addition to the environmental factors, genetics and/or immune status may also play a role in the malignant transformation. When mutations persist in proto-oncogenes, an uncontrolled rate of mitosis could lead to cell transformation and tumor formation. The levels of activating transcription factors in the nucleus of melanoma-inducing cells are associated with increased metastatic activity. 6 If early diagnosis is made, surgical excision is curative. However, once it metastasizes, the prognosis can be dismal, because metastatic melanoma does not response to chemotherapy, immunologic therapy, or radiotherapy. 7

Previous studies have demonstrated that VWF, FVIII, platelets, and other procoagulants, such as tissue factor, factor X, and thrombin, may not only result in the development of deep vein thromboembolism, but may also be associated with metastasis of malignant melanoma. 8 9 Plasma levels of VWF antigen are elevated in patients with metastatic melanoma, primarily resulting from activation of endothelium. 10 Depending on the experimental model, *vwf* -deficient mice are reported to exhibit either significantly increased or reduced metastatic potential. 11 12 Analysis of blood samples of metastatic melanoma patients demonstrated that increased plasma levels of procoagulant proteins such as VWF 13 and vascular endothelial growth factor A (VEGF-A) 13 and decreased expression or ADAMTS13 (a disintegrin and metalloprotease with thrombospondin type 1 repeats 13) activity create an environment that promotes tumor-associated thrombosis. 13

FVIII is an important procoagulant protein that binds to VWF with high affinity. In the coagulation cascade, thrombin activates FVIII to form FVIIIa, which dissociates from VWF and acts as a cofactor for factor IXa to activate factor X, which then activates prothrombin to form more thrombin. Thrombin cleaves fibrinogen to fibrin, which stabilizes platelets and clots. 14 Previous studies have demonstrated the association between baseline plasma FVIII activity in cancer patients, specifically in those with breast, colorectal, and cutaneous malignancies. 15 16 However, the association between the genetic alterations in the gene encoding *FVIII* , *VWF* , and *ADAMTS13* and long-term outcomes in patients with cutaneous melanoma has not been investigated in a large cohort of datasets.

This article takes advantage of the recent whole exome sequence (WES) datasets, mRNA expression (RNA-seq) data, and bioinformatics tools to determine the prognostic value of somatic mutations in *FVIII* , *VWF* , and *ADAMTS13* in cutaneous melanoma. Our results demonstrate that somatic mutations in *FVIII* , but not in *VWF* and *ADAMTS13* , are associated with a more favorable survival outcome. Further investigation of the molecular mechanism underlying how *FVIII* mutations are associated with a better outcome will help understand the pathogenesis of cutaneous melanoma, which may lead to the development of a novel therapeutic for such malignancy.

## Materials and Methods

### Data Mining with cBioPortal and TCGA Browser v0.9

Using cBioPortal, cross cancer studies were first performed to identify cancer studies with the highest mutation rates within *FVIII* , *VWF* , and *ADAMTS13* genes. Since the top-5 identified studies were all melanoma-related projects from different institutions, we focused on analyzing these three genes in melanoma datasets. All searches were performed according to cBioPortal's online instructions ( http://www.cbioportal.org/index.do ). The survival analysis related to mRNA expression of *VWF* was performed on the TCGA Browser 0.9 from the University Hospital Zurich ( http://tcgabrowser.ethz.ch:3839/TEST/ ). The mRNA expression of *VWF* was ranked from high to low, and the top one-third cases were defined as “high *VWF* ,” while the bottom one-third of cases were defined as “low *VWF* .”

### Melanoma Datasets for Analyses and Validation

As of June 4, 2017, the cBioPortal includes five melanoma datasets. The provisional “TCGA skin cutaneous melanoma” is the largest one with a total of 479 samples. Of these, 113 cases without mutation data were excluded from our study and the remaining 366 cases were used for further analyses. The corresponding clinical and pathological information associated with the patients in this dataset was downloaded from both the NCI's Genomic Data Commons ( https://gdc-portal.nci.nih.gov/ ) and the cBioPortal. The data from the two sources were compared to ensure that the most up-to-date clinical and pathological information was used for this analysis. The mutation pattern in *FVIII* , *VWF* , and *ADAMTS13* genes was found to be similar in the second cohort of 121 Broad patients 17 and the third cohort of 91 Yale melanoma patients. 18

### Selection of the Melanoma Diagnostic Features

The pathologic criteria used to determine any correlation with the mutations were based on the current WHO classification of human cutaneous melanoma and the AJCC cancer staging manual, 7th edition, including tumor depth (Clark's level and Breslow's thickness), tumor size, tumor site, lymph node, and metastasis status.

### Statistical Analysis

Clinical and pathological predictors in this study are listed in Table 1 . The primary outcome is the mortality rate. Fisher's exact test was used to compare the categorical variables between the group with *FVIII* mutations and those without *FVIII* mutations. The Student *t* -test was used to compare the continuous variables between the subgroups. Univariate analysis was performed using the log-rank test to correlate each clinical and pathological predictor with survival outcome. All tests were performed using the XLSTAT version 2014 (Addinsoft XLSTAT, France). All *p* -values were nonpaired and two sided. *p* -Values less than 0.05 and 0.01 are considered to be statistically significant and highly significant, respectively.

**Table 1:** Patient characteristics of categorical variables in *FVIII* -mutated and *FVIII* nonmutated groups

Variable	Categories	*FVIII* nonmutated ( *n* = 312)		*FVIII* mutated ( *n* = 54)		*p* -Value
		Number of cases	%	Number of cases	%	
Gender	Female	118	37.8	22	40.7	0.835
	Male	194	62.2	32	59.3	0.835
Race	Asian	9	2.9	1	1.9	0.641
	Black	1	0.3	0	0.0	0.671
	While	301	96.5	52	96.3	0.207
	Unknown	1	0.3	1	1.9	
Tumor site	Distant metastasis	6	1.9	0	0.0	0.295
	Extremities	134	42.9	20	37.0	0.312
	Head and neck	22	7.1	6	11.1	0.341
	Regional cutaneous a	3	1.0	4	7.4	**0.002**
	Regional lymph node	18	5.8	5	9.3	0.368
	Trunk	118	37.8	18	33.3	0.418
	Unknown	11	3.5	1	1.9	N/A
Current tumor status	**Tumor free**	**138**	**44.2**	**33**	**61.1**	**0.042**
	**With tumor**	**157**	**50.3**	**18**	**33.3**	**0.012**
	Unknown	17	5.4	3	5.6	N/A
Clark level at diagnosis	I	5	1.6	0	0.0	0.340
	II	13	4.2	2	3.7	0.836
	III	51	16.3	9	16.7	0.959
	IV	113	36.2	18	33.3	0.558
	V	40	12.8	3	5.6	0.109
	Unknown	90	28.8	22	40.7	N/A
Primary melanoma ulceration	No	104	33.3	16	29.6	0.484
	Yes	123	39.4	17	31.5	0.198
	Unknown	85	27.2	21	38.9	N/A
T	T0	15	4.8	7	13.0	**0.025**
	Tis	7	2.2	0	0.0	0.258
	T1	24	7.7	5	9.3	0.752
	T2	57	18.3	9	16.7	0.693
	T3	63	20.2	6	11.1	0.094
	T4	102	32.7	15	27.8	0.382
	TX	44	14.1	12	22.2	N/A
N	N0	149	47.8	31	57.4	0.295
	N1	54	17.3	7	13.0	0.373
	N2	36	11.5	7	13.0	0.837
	N3	41	13.1	4	7.4	0.207
	NX	32	10.3	5	9.3	N/A
M	M0	275	88.1	51	94.4	0.806
	**M1** b	**21**	**6.7**	**0**	**0.0**	**0.046**
	MX	16	5.1	3	5.6	N/A
Stage	Stage 0	6	1.9	0	0.0	0.295
	Stage I	50	16.0	10	18.5	0.733
	Stage II	80	25.6	16	29.6	0.646
	I/II/not sure	6	1.9	4	7.4	0.027
	Stage III	125	40.1	18	33.3	0.263
	Stage IV	18	5.8	1	1.9	0.215
	Unknown	27	8.7	5	9.3	N/A
Postoperative chemotherapy	No	210	67.3	44	81.5	0.093
	Yes	62	19.9	6	11.1	0.104
	Unknown	40	12.8	4	7.4	N/A
Postoperative radiation	No	287	92.0	50	92.6	0.503
	Yes	16	5.1	4	7.4	0.540
	Unknown	9	2.9	0	0.0	N/A

Abbreviations: M, distant metastases; N, lymph node; N/A, not determined; T, tumor; X after T, N, M indicates unknown status.

Values in bold indicate the difference between the two groups is statistically significant with *p* < 0.05.

a This category includes satellite and in-transit metastasis.

b All 21 metastatic cases were found in the *FVIII* nonmutated group.

## Results

### Mutations in FVIII, VWF, and ADAMTS13 in Various Malignant Neoplasms

**Fig. 1:**
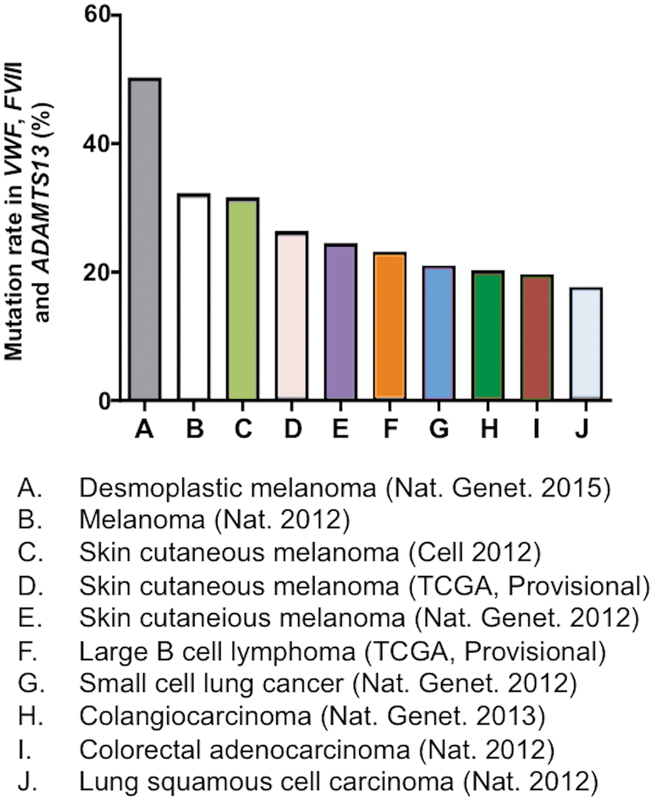
Top-10 cancer studies show the highest mutation rates in genes encoding *FVIII* , *VWF* , and *ADAMTS13* . The rates of *FVIII* , *VWF* , and *ADAMTS13* mutations were obtained by searching database among 126 cancer studies using cBioPortal. Each bar represents the percentage of mutation found in all three genes (e.g., *FVIII* , *VWF* , and *ADAMTS13* ) in each individual study.

The cBioPortal database has collected a large group of cancer genomic sequencing data. As of June 4, 2017, there were 25,719 samples from 126 cancer studies. In all cancer studies, the mutations in *FVIII* , *VWF* , and *ADAMTS13* were reported in 93 cancer studies. The top-5 studies were all melanoma studies from different institutions with the mutation rates ranging from 24 to 50%. 18 19 High mutation rates in *FVIII* , *VWF* , and *ADAMTS13* were also reported in diffuse large B cell lymphoma (22.9%), small cell lung carcinoma (20.7%), cholangiocarcinoma (20.0%), and colorectal adenocarcinoma (19.4%; Fig. 1 ).

### Characteristics of Patients with or without FVIII Mutations

In the provisional “TCGA skin cutaneous melanoma” dataset, 54 patients had *FVIII* mutations and 312 patients had no *FVIII* mutation. All mutations found by WES were confirmed by RNA-seq in the same specimen, which were not present in the genomic DNA isolated from the patient leukocytes. 17 19 No significant difference was identified between patient's age, gender, Clark's level, Breslow's thickness, primary tumor ulceration, lymph node status, stage, overall copy number variation, and the percentage of postoperative chemo/radiation therapy in melanoma patients with and without *FVIII* mutations except for mutations counts ( Table 1 and Supplementary Table 1 ). However, Fisher's exact test demonstrated that all 21 cases (6.7%) with metastasis were all reported in the *FVIII* nonmutated group, but no metastatic case (0%) was reported in the *FVIII* -mutated group ( *p* = 0.046). Furthermore, patients with the *FVIII* mutation are more likely to be in the tumor-free group (61.1 vs. 44.2%, *p* = 0.042; Table 1 ). Interestingly, the overall mutation counts were also significantly higher in the *FVIII* -mutated group than in the *FVIII* nonmutated group ( *p* = 0.013; Supplementary Table 1 ).

### Mutation Profiles in FVIII, VWF, and ADAMTS13 in Cutaneous Melanoma

**Fig. 2:**
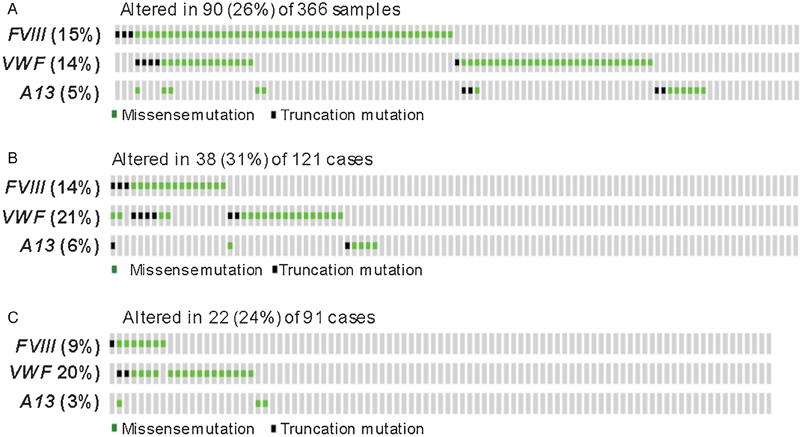
Mutation profiles of *FVIII, VWF* , and *ADAMTS13* found in cutaneous melanoma of three datasets. ( **A** ) TCGA provisional; ( **B** ) broad cell, 2012; and ( **C** ) Yale Nature Genetics, 2012.

**Fig. 3:**
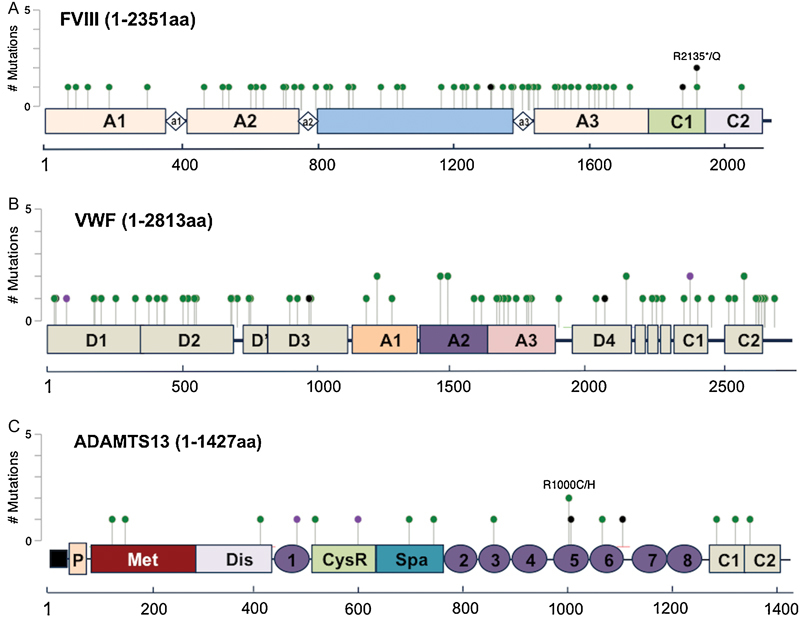
Detailed mutation maps of *FVIII* , *VWF* , and *ADAMTS13* found in patients with cutaneous melanoma. Each dot above the protein molecule represents a mutation, which spreads across the entire encoded protein of FVIII ( **A** ), VWF ( **B** ), and ADAMTS13 ( **C** ). There is no evidence of a “hotspot” of gene mutations.

Among five melanoma studies, the provisional “TCGA skin cutaneous melanoma” project was the largest one with sequencing data from 479 samples. This was also the only cohort with corresponding pathological and clinical information described earlier for each patient. The mutation rates in *FVIII* , *VWF* , and *ADAMTS13* from the TCGA provisional datasets were 15, 14, and 5%, respectively ( Fig. 2A ). Similar results were obtained from other two datasets: 121 Broad patients ( Fig. 2B ) 17 and 91 Yale patients 18 ( Fig. 2C ). A majority of mutations identified were missense and nonsense resulting in amino acid changes and a truncation of FVIII protein, respectively. The number of mutations in *FVIII* , *VWF* , and *ADAMTS13* appeared to directly correlate with the length of coding regions of the genes ( *r* ^2^ = 0.993). However, there was no evidence of a mutational hotspot in any of these three genes ( *FVIII* , *VWF* , and *ADAMTS13* ; Fig. 3 ). All these gene mutations found by WES were verified with the RNA-Seq analysis in the same tumor samples, which were not present in the genomic DNA isolated from leukocytes of the patients. 17 18 19 These results suggest the somatic mutation, rather than inherited. These mutated gene products were expressed in the tumor cells. Furthermore, there was a strong tendency of coexistence of mutations in both *FVIII* and *VWF* or in both *VWF* and *ADAMTS13* in a single patient (not shown).

### Effect of Somatic Mutations in FVIII, VWF, and ADAMTS13 on the Overall Survival of Melanoma Patients

**Fig. 4:**
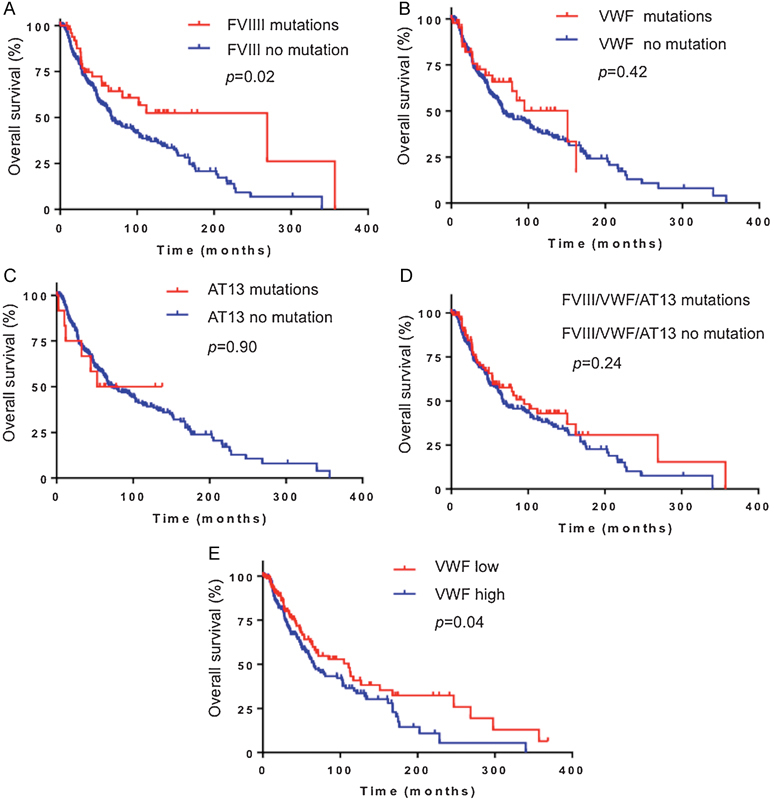
Association of mutations in *FVIII* , *VWF* , and *ADAMTS13* and their expression levels with the long-term outcomes of cutaneous melanoma. ( **A** ) *FVIII* mutations are associated with a better overall survival rate ( *p* = 0.02). ( **B** and **C** ) *VWF* and *ADAMTS13 (AT13)* mutations are not associated with a better overall survival rate ( *p* = 0.42 and *p* = 0.90, respectively). ( **D** ) Combined mutations of *VWF* / *FVIII* / *AT13* are not associated with an overall survival rate ( *p* = 0.24). ( **E** ) Low *VWF* mRNA expression is associated with a better survival rate ( *p* = 0.04).

Log-rank test showed that mutations in *FVIII* ( Fig. 4A ), but not in *VWF* ( Fig. 4B ) and *ADAMTS1* 3 ( Fig. 4C ), were significantly associated with a better survival rate in melanoma patients. In addition, no association was found between the combined mutations of *VWF* , *FVIII* , and *ADAMTS13* with the long-term survival rate ( Fig. 4D ). Interestingly, low *VWF* , but not *FVIII* and *ADAMTS13* , mRNA expression in the melanoma tissue itself was associated with a better overall survival rate ( *p* = 0.02; Fig. 4E ), suggesting that the decreased *VWF* gene expression in association with *FVIII* mutations may have a protective effect in patients with melanoma. Specifically, patients with the *FVIII* mutations had a median survival of 269 months compared with the median survival of 67 months in those without the *FVIII* mutations ( *p* = 0.02).

### The Effect of Mutations on mRNA and Protein Expressions of FVIII, VWF, and ADAMTS13 in Melanoma Patients

**Fig. 5:**
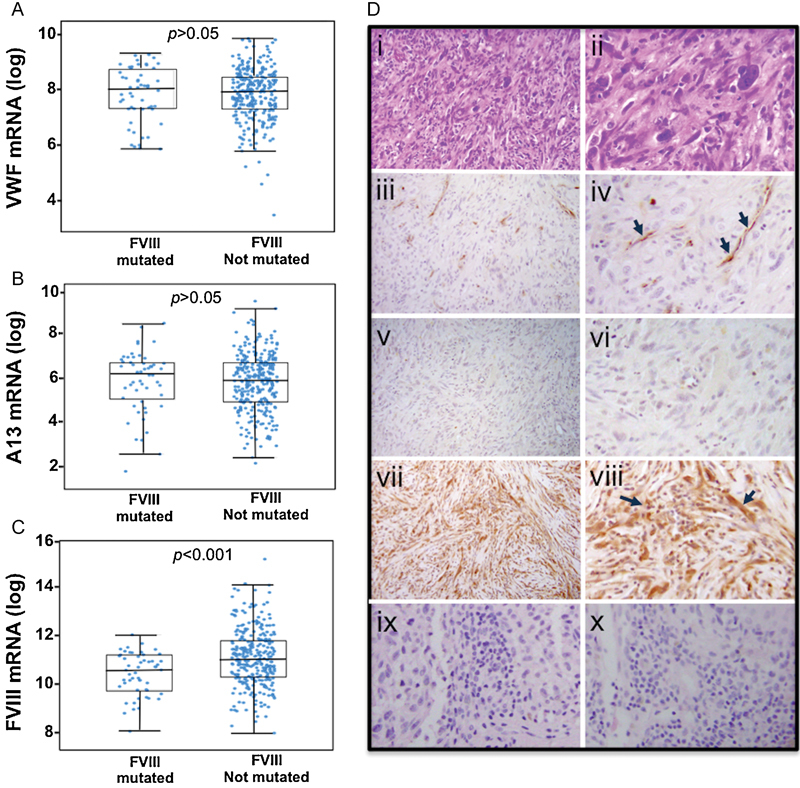
Association between mutations in *FVIII* and expression of *VWF* , *FVIII* , and *ADAMTS13* mRNA in patients with cutaneous melanoma. The levels of mRNA expression (mean ± 95% confidential interval) for *VWF* ( **A** ), *FVIII* ( **B** ), and *ADAMTS13* ( **C** ) in patients with or without *FVIII* mutations. ( **D** ). Hematoxylin and eosin staining (i and ii) and immunohistochemistry with rabbit anti-VWF IgG (iii and iv), sheep anti-FVIII IgG (v and vi), and rabbit anti-ADAMTS13 IgG (vii and viii), followed by streptavidin-peroxidase conjugated anti-rabbit IgG or anti-sheep IgG or secondary antibody only (ix and x).

To investigate the possible underlying mechanism of the protective role of *FVIII* mutations in patients with cutaneous melanoma, we tested the effect of mutations in *FVIII* , *VWF* , and *ADAMTS13* on mRNA expression based on the RNA-Seq data. As shown, *FVIII* mutations did not result in a significant alteration of *VWF* ( Fig. 5A ) and *ADAMTS13* mRNA expression ( Fig. 5B ), but they did significantly lower the *FVIII* mRNA expression ( *p* < 0.001) as one might anticipate ( Fig. 5C ). We further tested the entire mRNA expression profile in the tumor tissue from patients with *FVIII* mutations. Using both *p* -value (Student's *t* -test) and Q-value (false discovery rate) less than 0.05 as the inclusion criteria, we identified a total of 44 genes with a significant difference between those with the *FVIII* mutations and those without ( Supplementary Table 2 ). A total of 38 genes showed an increased expression of mRNA in the *FVIII* -mutated group and 6 genes, including *VWF* , showed decreased mRNA expression in *FVIII* -mutated group compared with *FVIII* nonmutated group ( Supplementary Table 2 ).

VWF is produced in all vascular endothelium; FVIII is expressed only in the sinusoidal and pulmonary vascular endothelium; ADAMTS13 is primarily synthesized in hepatic stellate cells, 20 but also found in trace amount in the vascular endothelium 21 22 and platelets. 23 While *VWF* , *FVIII* , and *ADAMTS13* mRNAs were identified in the melanoma tissues through RNA-Seq analysis, the protein expression has never been examined in tumor tissues. We set to determine the protein expression in seven melanoma cases by immunohistochemistry. The results showed that VWF ( Fig. 5D , iii and iv) and FVIII ( Fig. 5D , v and vi) were not detected in the melanoma cells, but ADAMTS13 immune reactivity was strongly positive in melanoma cells ( Fig. 5D , vii and viii). Control staining was negative when primary antibodies were omitted ( Fig. 5D , ix and x). The specific VWF staining was detected in the vascular endothelium within the melanoma tissues ( Fig. 5D , iii and iv, arrowheads). These results demonstrate that while mRNAs for *VWF* , *FVIII* , and *ADAMTS13* are all expressed in melanoma tissue, the protein concentrations of VWF and FVIII, but not ADAMTS13, in the tumor tissues may be quite low.

## Discussion

This study highlights the high mutation rates in genes encoding *FVIII* , *VWF* , and *ADAMTS13* in patients with metastatic cutaneous melanoma ( Figs. 1 2 3 ). The presence of mutations in *FVIII* , but not in *VWF* and *ADAMTS13* or a combined mutation rate of *FVIII, VWF* , and *ADAMTS13* , is associated with a more favorable overall survival in these patients ( Fig. 4 ). The results suggest that the mutation burden in *FVIII* may be useful as a prognostic marker. These results may also suggest a potential role of FVIII protein and its carrier protein VWF in the pathobiology of melanoma including tumor growth and metastasis by affecting the tumor microenvironment.

As shown in our data, patients with the *FVIII* mutations exhibited significantly lower expression of the *FVIII* mRNA, which may be translated with the reduced levels of FVIII procoagulant activity in situ around the tumors ( Fig. 5 ), although immunohistochemical studies did not detect the FVIII protein in all seven cases of melanoma ( Fig. 5 and Table 2 ). This may be caused by the low abundance of FVIII protein in any given cell. Further determination of FVIII synthesis and secretion from cultured melanoma cells with or without *FVIII* mutations may help better understand the pathophysiological relevance of the *FVIII* mutations.

**Table 2:** Expression of VWF, FVIII, and ADAMTS13 in melanoma tissues by immunohistochemistry

Case no.	Age at diagnosis (y)	Gender	Diagnosis	Location	Clark level	Breslow depth	Melanoma		
							FVIII	VWF	ADAMTS13
1	85	Male	Recurrent melanoma	Face	N/A	N/A	(−)	(−)	(+++)
2	63	Female	Melanoma	Right arm	N/A	N/A	(−)	(−)	(−)
3	69	Female	Invasive melanoma	Left thigh	III	0.8 mm	(−)	(−)	(++)
4	35	Female	Invasive melanoma	Left abdomen	II	0.38 mm	(−)	(−)	(+)
5	64	Female	Melanoma	Right parietal scalp	V	4.15 mm	(−)	(−)	(++)
6	84	Male	Invasive melanoma	Left cheek	III	0.45 mm	(−)	(−)	(++)
7	67	Male	Invasive melanoma	Right lateral chest		0.65 mm	(−)	(−)	(++)

Abbreviations: ADATMS13, a disintegrin and metalloprotease with thrombospondin type 1 repeats 13; FVIII, factor VIII; N/A, not available; VWF, von Willebrand factor.

Note: −, +, ++, and +++ denote negative, mild, moderate, and strong positive in staining on tumor cells. VWF antibody stained all vessels.

The role of FVIII in melanoma biology has been previously investigated in animal studies. Hemophilia A mice lacking plasma FVIII activity were protective against the formation of lung metastatic foci after an intravenous inoculation of a murine melanoma cell line (B16F10). 24 25 A single-dose infusion of human FVIII (100 U/kg) into hemophilia A mice significantly enhanced the lung metastasis of murine melanoma cells. 25 In contrast, administration of a factor Xa inhibitor (i.e., tinzaparin) to wild-type mice 26 or a direct thrombin inhibitor (i.e., lepirudin or argatroban) into wild-type mice 27 and hemophilia A mice 24 25 significantly reduced lung metastasis, suggesting that thrombin generation in the presence or absence of FVIII contributes to pulmonary metastasis. Thrombin is known to support tumor spreading and metastasis, predominantly mediated by the cleavage of a protease-activated receptor-1 (PAR-1), which is highly expressed on the surface of platelets, endothelial cells, and metastatic cancer cells. 28 29

The role of VWF and its cleaving protease ADAMTS13 in melanoma metastasis is much less clear. While mutation rates in *VWF* are not associated with the long-term outcome in patients with melanoma, its plasma levels or the presence of ultra-large VWF on endothelial surface is shown to be important for the development of thrombosis and tumor metastasis. 12 13 30 31 VWF is highly expressed in the vascular endothelium within the tumor, but not in the malignant melanoma cells themselves ( Fig. 5 and Table 2 ). Therefore, VWF may be an important adhesion protein involving in thromboembolism and tumor metastatic process. Consistent with this notion, patients with melanoma had approximately twofold increase in their mean plasma concentrations of VWF antigen compared with the healthy controls (24 vs. 14 μg/mL). 13 The increased levels of plasma VWF correlated with enhanced tumor progression in these patients. 32 Blocking antibodies against VWF appeared to attenuate the binding of tumor cells to platelets by 75 to 81%. 30 Paradoxically, lung colonization by cancer cells was enhanced in the *VWF* -knockout mice 12 and inhibition of platelet glycoprotein Ibα led to significant increase in the formation of pulmonary foci of tumor cells. 33 While the mutation burden in ADAMTS13 is not associated with the long-term outcome in patients with melanoma, reduced ADAMTS13 activity was reported in the tumor microenvironment or vessels, 13 likely resulting from the locally released inflammatory cytokines (TNF-α, IFN-γ, and IL-6), which may inhibit ADAMTS13 synthesis 21 and activity. 34 In seven cases we studied by immunohistochemistry, ADAMTS13 expression varied significantly from negative to strong positive ( Fig. 5 and Table 2 ), although proteolytic activity of tissue-derived ADAMTS13 was not determined. Reduced ADAMTS13 activity may result in an accumulation of ultra-large VWF on endothelial surface and heightened platelet adhesion and aggregation, promoting thromboembolism and metastasis.

We conclude that high mutation rates in *FVIII* , but not in *VWF* and *ADAMTS13* , may have a value in predicting a long-term outcome in patients with cutaneous metastatic melanoma. The findings demonstrate a potential role of procoagulant factors in pathobiology of metastatic melanoma, providing a rationale for a novel therapeutic strategy in patients with metastatic melanoma.
