# Supplementary material for: Mutations in Coagulation Factor VIII Are Associated with More Favorable Outcome in Patients with Cutaneous Melanoma
Source: TH Open. 2017 Oct 20;1(2):e113–21. doi: 10.1055/s-0037-1607337 (PMC5690574; doi:10.1055/s-0037-1607337)
Supplement: Supplementary file 1 — Supplementary Tables [file 10-1055-s-0037-1607337-s170005.pdf]

Supplementary Tables

Table S1 Patient characteristics of continuous variables in FVlll-mutated and FVlll nonmutated groups

**Characteristics**	***FVIII* nonmutated (mean ± SD)**	***FVIII* mutated (mean ± SD)**	***p*-Value[a]**
Diagnosis age	57 ± 16	57 ± 16	0.918
Breslow's thickness at diagnosis	4.9 ± 7.6	5.6 ± 5.2	0.523
Overall mutation count	381 ± 443	1,148 ± 2,162	**0.013**
Overall CNV percentage	31 ± 20%	32 ± 22%	0.738

Abbreviations: CNV, copy number variation; SD, standard deviation.

[a]*p*-Values were calculated by Student's *t*-test. *p*-Value less than 0.05 in bold is considered to be statistically significant.

Table S2 A total of 44 genes with significant difference in patients with *FVIII* mutated versus *FVIII* nonmutated

**Increased mRNA expression in FVlll-mutated patients[a] (38 genes)**							**Decreased mRNA expression in FVlll-mutated patients[a] (6 genes)**
FOSL1	POLE2	CCNB2	PNPT1	LCLAT1	WDR12	DARS	GPR146
TMEM108	C150RF41	TIPIN	PRELID3B	ARPP19	PPP2R3C	SUM01P3	ARHGEF15
DEPDC1	ARL61P6	ZWILCH	PLEKHA3	DCAF17	SRI		VWF
MLF1	RPL23AP82	DTWD1	HAUS2	DNAJC24	MTX2		CTSL
ANLN	KATNBL1P6	SUPT3H	METTL5	STYX	IFT52		ITGA11
CDKN3	SNAPC1	CSORF34	OSGEPL1	C10RF109	FARSB		HPGD

[a]Both *p*-value (Student's *t*-test) and Q-value (false discovery rate) <0.05 as the inclusion criteria.
